# Biological Evaluation of Oral Care Products Using 3D Tissue-Engineered In Vitro Models of Plaque-Induced Gingivitis

**DOI:** 10.3390/dj12050126

**Published:** 2024-05-06

**Authors:** Emilia Barker, Lina AlQobaly, Zahab Shaikh, Kirsty Franklin, Johanna Thurlow, Behfar Moghaddam, Jonathan Pratten, Keyvan Moharamzadeh

**Affiliations:** 1 School of Clinical Dentistry, University of Sheffield, Sheffield S10 2TA, UK; emilia.barker@sheffield.ac.uk (E.B.); lina.alqobaly@gmail.com (L.A.); zahabnoshad@yahoo.com (Z.S.); k.l.franklin@sheffield.ac.uk (K.F.); j.thurlow@sheffield.ac.uk (J.T.); 2 Haleon, Weybridge, Surrey KT13 0DE, UK; behfar.x.moghaddam@haleon.com (B.M.);jonathan.r.pratten@haleon.com (J.P.); 3 Hamdan Bin Mohammed College of Dental Medicine, Mohammed Bin Rashid University of Medicine and Health Sciences, Dubai 505055, United Arab Emirates

**Keywords:** biocompatibility, oral mucosa, tissue engineering, inflammation, bacteria, mouthwash, toothpaste

## Abstract

Background: The aim of this study was to investigate and visualize the anti-inflammatory and anti-bacterial effects of different oral care products using an infected and inflamed 3D tissue-engineered gingival mucosal model. Methods: A 3D full-thickness oral mucosal model was engineered inside tissue culture inserts using collagen hydrogels populated with human gingival fibroblasts and THP-1 monocytes and layered with oral epithelial cell lines. Oral saliva bacteria were cultured and added to the surface of the models and inflammation was further simulated with lipopolysaccharide (LPS) of *Escherichia coli*. The 3D models were exposed to three different types of toothpastes, a chlorhexidine antiseptic mouthwash, different antibiotics, and a mechanical rinse with phosphate-buffered saline (PBS) prior to biological evaluation using the PrestoBlue tissue viability assay, histology, optical coherence tomography (OCT), confocal microscopy, and measurement of the release of the inflammatory markers IL-1β, IL-6, and IL-8 with ELISA. Results: Multiple-endpoint analyses of the infected oral mucosal models treated with different anti-bacterial agents showed consistent outcomes in terms of tissue viability, histology, OCT, and confocal microscopy findings. In terms of anti-inflammatory testings, the positive control group showed the highest level of inflammation compared with all other groups. Depending on the anti-bacterial and anti-inflammatory potential of the test groups, different levels of inflammation were observed in the test groups. Conclusions: The inflamed 3D oral mucosal model developed in this study has the potential to be used as a suitable in vitro model for testing the biocompatibility, anti-inflammatory, and anti-bacterial properties of oral care products including mouthwashes and toothpastes. The results of this study indicate that the chlorhexidine mouthwash has both anti-bacterial and cytotoxic effects on the 3D oral mucosal model. Hyaluronic-acid-containing toothpaste has significant anti-bacterial and anti-inflammatory effects on the 3D oral mucosal model.

## 1. Introduction

In vivo animal models have frequently been used for investigating the immune response and host–pathogen interactions. However, application of these models is limited by legal and ethical restrictions and there are additional technical challenges in testing oral care products, such as toothpastes and mouthwashes, using animals where a controlled biological system and consistent exposure protocols are required [1].

In vitro monolayer cell culture systems have been developed and used extensively for biocompatibility testing in dentistry [2,3,4] as well as assessments of the cytotoxicity and safety of different oral care products [5]. The main limitation of in vitro monolayer cell culture test systems for evaluation of the mucosal biocompatibility of oral care products is that they may not fully represent the clinical situation as they lack the barrier function of a human oral mucosa [6].

The development of novel three-dimensional (3D) tissue-engineered human oral mucosal models is a breakthrough in the tissue engineering of oral tissues due to the high degree of resemblance of the 3D models to the native tissues compared with monolayer systems [7,8], and they provide more realistic qualitative and quantitative data in relation to biological interactions between dental biomaterials and a human oral mucosa. These 3D tissue models have been validated by comparing them with fresh clinical biopsies in terms of the biological response to different stimuli and have recently been used to investigate the bacterial invasion of a human oral mucosa and quantify the release of various pro-inflammatory cytokines and chemokines [9].

Advanced 3D models of a combination of a human oral mucosa and alveolar bone have also been developed using different conventional scaffolds and 3D-printed systems [10]. These models can potentially be used in in vitro 3D test systems in order to reduce the need for animal testing and to assess the biological interactions of the oral tissues with different biomaterials. Other potential applications include vascularization studies and the investigation of hard and soft oral tissue pathologies, disease progression and treatment, as well as the assessment of different drug delivery systems [11].

Tissue-engineered 3D models of a human oral mucosa have also been used in recent years for the biological assessment of dental materials [12], to evaluate gingival soft-tissue attachment to different implant and abutment surfaces made of ceramic, metal, and plastic [13,14], and for studying the biological interactions between a human oral mucosa and electronic cigarettes [15].

Biocompatibility and safety issues regarding different types of oral care products, such as toothpastes and mouthwashes, and their ingredients have been thoroughly reviewed previously [16]. However, there are no in vitro studies comparing the anti-inflammatory and anti-bacterial effects of different oral care products using 3D tissue-engineered in vitro models of plaque-induced gingivitis.

The aim of this study was to assess the anti-inflammatory and anti-bacterial effects of different oral care products using infected and inflamed 3D tissue-engineered oral mucosal models. The objectives of this study were to assess the effects of the removal of the oral bacterial biofilm on the reduction in the inflammation of the 3D oral mucosal model and assess the anti-bacterial effects of different toothpastes using a model of saliva bacteria cultured on a non-vital mucosal matrix.

## 2. Materials and Methods

### 2.1. Cell Source

Normal human oral fibroblast cells were obtained from the biorepository at the School of Clinical Dentistry, University of Sheffield. They had previously been harvested with written informed consent from healthy adult patients having oral surgery procedures, such as wisdom tooth removal and implant surgery, at Charles Clifford Dental Hospital, and the appropriate ethical permission was obtained from the National Research Ethics Services Committee—UK (Ethics reference number 15/LO/0116, Protocol Number: STH18551, Date of approval: 21 January 2015).

Oral keratinocyte cells (the TR146 cell line) were gifted to us by Cancer Research UK, and we also explored the feasibility of using the immortalized OKF6/TERT-2 human oral keratinocyte cell line, which was kindly provided by Brigham and Women’s Hospital, Harvard Institute of Medicine, USA.

The THP-1 monocyte cell line was obtained from Culture Collections, Public Health England, Porton Down, Salisbury, SP4 0JG, UK.

### 2.2. Cell Culture

Oral fibroblasts and keratinocytes were cultured in Dulbecco’s modified Eagle’s medium (DMEM) (Sigma, Dorset, UK), supplemented with 10% fetal calf serum (FCS) (GIBCO, ThermoFisher Scientific, Loughborough, UK), 100 IU:100 mg/mL penicillin–streptomycin (Sigma, Dorset, UK), and 2 mM L-glutamine (Sigma, Dorset, UK).

THP-1 monocytes were grown in the RPMI 1640 culture medium (Sigma, Dorset, UK) supplemented with 10% FCS and 2 mM L-glutamine.

Cultures were kept in 5% CO_2_ incubators at 37 °C. The cells were cultured to 80–100% confluency.

### 2.3. 3D Oral Mucosa Models

A solution of 5 mg/mL rat-tail type I collagen (R & D system, Minneapolis, MN, USA), concentrated DMEM (×10), 8.5% FCS (*v*/*v*), 2 mM L-glutamine 2, and reconstitution buffer including 20 mM 4-(2-hydroxyethyl)-1-piperazineethanesulfonic acid and 22 mg/mL sodium bicarbonate was prepared and neutralized by a 1 M sodium hydroxide solution to pH = 7.4 at a cold temperature by keeping the solution on ice. Collagen hydrogels were mixed with fibroblasts (5 × 10^5^/mL) and THP-1 monocytes (1 × 10^5^ /mL), and 1 mL of the cell-containing collagen mixture was transferred to Millipore tissue culture inserts with a 0.4 µm pore size, incubated at 37 °C for 2 h for solidification, and then submerged in complete DMEM for 3 days.

A total of 1 × 10^6^ keratinocytes were seeded onto the surface of each model and further cultured in a submerged condition for 3 days. The oral mucosal models were then raised to an air–liquid interface and further cultured for 7 days.

### 2.4. Saliva Bacteria Culture and Addition to 3D Oral Mucosa Model

The saliva sample was collected from a healthy non-smoker with informed consent under ethical approval from the UK Health Research Authority (Ethics reference number 18/WM/0068, Protocol Number: STH20225, Date of approval: 4 April 2018). Saliva bacteria were cultured overnight in Brain Heart Infusion (BHI) broth (Oxoid) containing brain infusion solids, beef heart infusion solids, proteose peptone, glucose, sodium chloride, and di-sodium phosphate at 37 °C. On the following day, the bacteria were counted, and the concentration was adjusted to 20 × 10^7^ per ml. A total of 50 µL of the bacterial suspension (1 × 10^7^) in BHI medium was applied to the epithelial surface of each oral mucosa model and incubated overnight at 37 °C in antibiotic-free cell culture medium. Lipopolysaccharide (LPS) of *Escherichia coli* (*E. coli*) (Sigma-Aldrich, Gillingham, UK) was also added to the culture medium at a 10 μg/mL concentration to further simulate the inflammatory condition in the 3D oral mucosal models.

### 2.5. Test Materials and Groups

The test groups were as follows:

1- A high-molecular-weight hyaluronic-acid-containing toothpaste (non-commercial) diluted in PBS at a 20% (*v*/*v*) concentration;
2- A fluoride-containing toothpaste (Aquafresh, GSK, Weybridge, UK) diluted in PBS at a 20% (*v*/*v*) concentration;
3- A 67% sodium-bicarbonate-containing toothpaste (Corsodyl, GSK, Weybridge, UK) diluted in PBS at a 20% (*v*/*v*) concentration;
4- The antibiotics penicillin and streptomycin (100 IU:100 mg/mL) (Sigma, Dorset, UK);
5- A 0.2% *w*/*v* chlorhexidine digluconate mouthwash (Corsodyl, GSK, Weybridge, UK);
6- Mechanical rinsing with phosphate-buffered saline (PBS) using a pipette;
7- No bacterial removal;
8- Oral mucosal models without bacteria.

### 2.6. Exposure Protocol

The test materials were added to the epithelial–biofilm surface of the oral mucosal models and were agitated for 2 min using an IKA Vibrax VXR orbital shaker (Janke & Kunkel GabH & Co. KG, Staufen, Germany) at 200 rpm. The models were then rinsed with PBS three times to wash off the test agents. In parallel, the infected models without any treatment were used as the positive control (no-removal) group and the uninfected models in sterile (bacteria-free) medium served as the negative control (no-bacteria) group (N = 4). Following the exposure and rinsing steps, the complete culture medium without antibiotics was added and the models were incubated for 72 h in a humidified atmosphere at 37 °C prior to multiple-endpoint analyses.

### 2.7. Biological Evaluation

#### 2.7.1. PrestoBlue Tissue Viability Test

The tissue viability of the oral mucosal models was measured using the PrestoBlue assay. The 10% PrestoBlue solution (Biosource, Camarilo, CA, USA) in DMEM was added to the 3D tissue models inside wells and the plates were then maintained in a CO_2_ incubator at 37 °C for 60 min. Triplicate 100 µL samples were then taken from each well and were transferred into a 96-well plate. Blank 10% PrestoBlue solution was also used as an additional control group. The fluorescence intensity was read using a fluorescent plate reader (Infinite 200 PRO TECAN, Mannedorf, Switzerland) at a 530 nm excitation wavelength and a 590 nm emission wavelength.

#### 2.7.2. Histological Processing

One 3D oral mucosal model from each group was processed for histological evaluation. The models were fixed in 10% (*v*/*v*) formalin solution for 24 h, and the specimens were processed for paraffin wax sectioning. Five-micrometer sections were prepared and stained with hematoxylin and eosin (H&E). Histological slides were examined by two observers independently blinded to the type of treatment that the tissue had received using an inverted microscope equipped with a digital camera. Multiple random pictures of the sections were obtained from the center of each model. If there were any discrepancies in the histological evaluation of the specimens between investigators, the differences were resolved by discussing them and reaching a consensus before removing the blindfold. Evaluation criteria included the continuity and thickness of the epithelial layer as well as the morphology and presence of cells in the connective tissue layer.

#### 2.7.3. Optical Coherence Tomography (OCT)

One 3D oral mucosal model from each group was scanned and analyzed using an OCT machine (RS-330, NIDEK, Gamagori, Japan) with colorative imaging to demonstrate any significant changes in the epithelial and connective tissue layers of the 3D oral mucosal models. The images were examined by two observers independently as described in Section 2.7.2 above.

#### 2.7.4. Confocal Microscopy

One 3D oral mucosal model from each group was used for confocal microscopy. The models were first rinsed with PBS three times and then a mixture of fluorescent labels, red propidium iodide (PI), and green CMFDA (Molecular Probes) in serum-free medium, both at a final concentration of 5 µM, was added to the oral mucosal models from each group and incubated for 1 h, after which the medium was discarded and the models were rinsed with PBS and fixed with 10% formalin solution (Sigma-UK). The analysis was carried out using a Zeiss LSM510 Meta confocal microscope at the confocal imaging facility at the Kroto Institute, University of Sheffield, with a set of two lasers (green, 488 nm and red, 543 nm). The images were examined by two observers independently as described in Section 2.7.2 above.

#### 2.7.5. Enzyme-Linked Immunosorbent Assay (ELISA)

Tissue culture supernatants were collected from all wells in each group (N = 4) and stored in a freezer for analysis for the presence of inflammatory cytokines. Tissue culture supernatants were analyzed for the presence of IL-1β, IL-6, and IL-8 using ELISA kits (Sigma, Dorset, UK) following the manufacturer’s instructions.

### 2.8. Anti-Bacterial Testing

To assess the anti-bacterial effects of the different test agents on saliva bacteria cultured on non-vital mucosal matrices (to eliminate the influence of live cells on the data), 6 mm punch biopsies of a non-vital porcine oral mucosa were obtained using the frozen porcine jaws stored in the laboratories at the School of Clinical Dentistry. Mucosal discs were washed with PBS three times, and 50 µL of the saliva bacteria suspension (1 × 10^7^) in BHI medium was applied to the surface of each substrate and incubated overnight at 37 °C in full-cell culture medium.

The sample size for this experiment was 5 mucosa biopsies per group (N = 5).

The test materials were added to the biofilm surface of the discs and were agitated for 2 min using a shaker as described above. The discs were then rinsed with PBS three times to wash off the test agents. The infected discs without any treatment were used as the positive control (no-removal) group and the uninfected sterile discs in bacteria-free medium served as the negative control (no-bacteria) group. Following the exposure and rinsing steps, the complete culture medium without antibiotics was added and the models were incubated in a humidified atmosphere at 37 °C for 72 h. A PrestoBlue assay with three samples from each well and confocal microscopy analysis were then carried out according to the protocol described in previous sections.

### 2.9. Statistical Analysis

Statistical analysis of the data was conducted with one-way ANOVA and Tukey’s multiple comparison test using the Minitab statistical analysis software version 21.1.0 (Minitab Inc.,University Park, PA, USA). *P*-values of less than 0.05 were considered statistically significant.

### 2.10. Pilot Study

In addition to the authors’ group’s previous studies [4,6] in the field of biocompatibility assessment of dental materials using 3D tissue-engineered oral mucosal models, a pilot study was also conducted to optimize the methods and further ensure their reliability and validity

## 3. Results

### 3.1. Tissue Viability Assay

**Figure 1:**
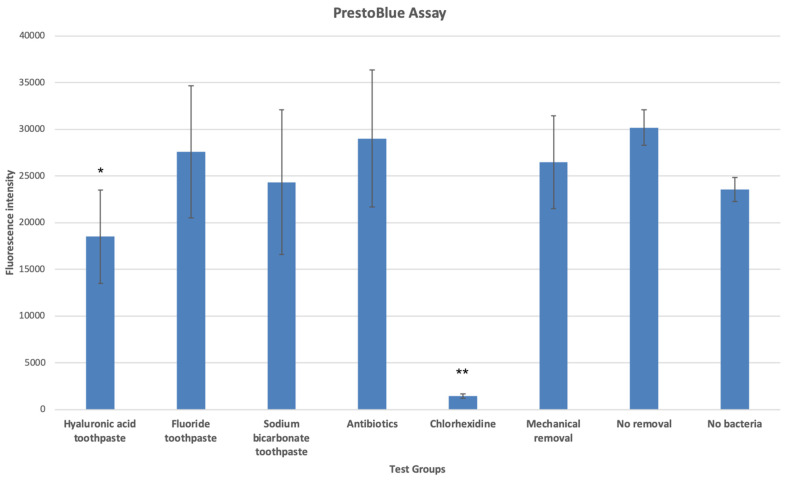
Tissue viability of 3D oral mucosal models in different test groups as assessed by the PrestoBlue assay. *, statistically significant difference (*p* < 0.05). **, statistically significant difference (*p* < 0.01).

The results of the PrestoBlue tissue viability assay are presented in Figure 1. Infected oral mucosal models treated with a chlorhexidine mouthwash and a hyaluronic acid toothpaste showed statistically significantly lower viability compared with the untreated infected 3D oral mucosal model group (*p* < 0.05). The chlorhexidine group also had statistically significantly lower viability compared with the non-infected oral mucosal model group (*p* < 0.05).

### 3.2. Histology

**Figure 2:**
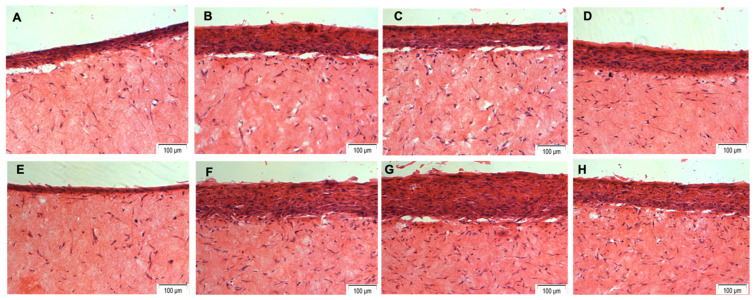
Microscopic views of H&E-stained histological sections of 3D tissue-engineered normal oral mucosal models in different test groups, including (**A**) a hyaluronic acid toothpaste, (**B**) a fluoride toothpaste, (**C**) a sodium bicarbonate toothpaste, (**D**) antibiotics, (**E**) chlorhexidine, (**F**) mechanical removal, (**G**) no removal, and (**H**) no bacteria. Original magnification, ×10.

Microscopic views of the histological sections of the 3D tissue-engineered oral mucosal models in different test groups are presented in Figure 2, which shows a multi-layered stratified epithelium and fibroblasts with monocytes within the connective tissue layer in all groups except for the inflamed oral mucosal model treated with chlorhexidine, which shows a very thin epithelial layer and a reduced number of fibroblasts within the connective tissue layer.

### 3.3. Optical Coherence Tomography (OCT)

**Figure 3:**
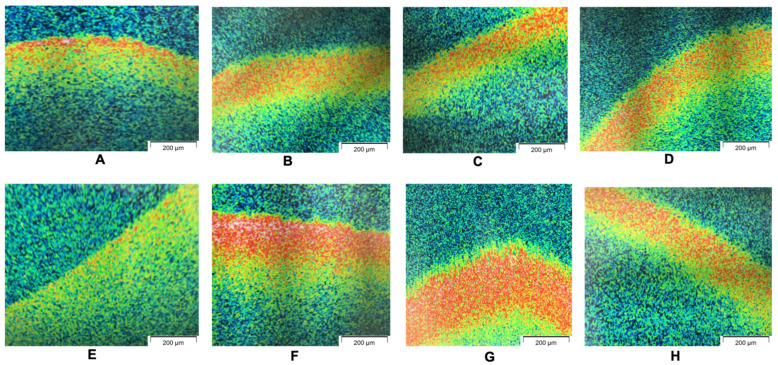
OCT images of 3D tissue-engineered normal oral mucosal models in different test groups, including (**A**) a hyaluronic acid toothpaste, (**B**) a fluoride toothpaste, (**C**) a sodium bicarbonate toothpaste, (**D**) antibiotics, (**E**) chlorhexidine, (**F**) mechanical removal, (**G**) no removal, and (**H**) no bacteria.

OCT views of the 3D oral mucosal models in different test groups are presented in Figure 3. OCT visualized the superficial layers of the mucosa. However, the junction between the epithelium and the connective tissue layer was not as clear as the histology images.

### 3.4. Confocal Microscopy

**Figure 4:**
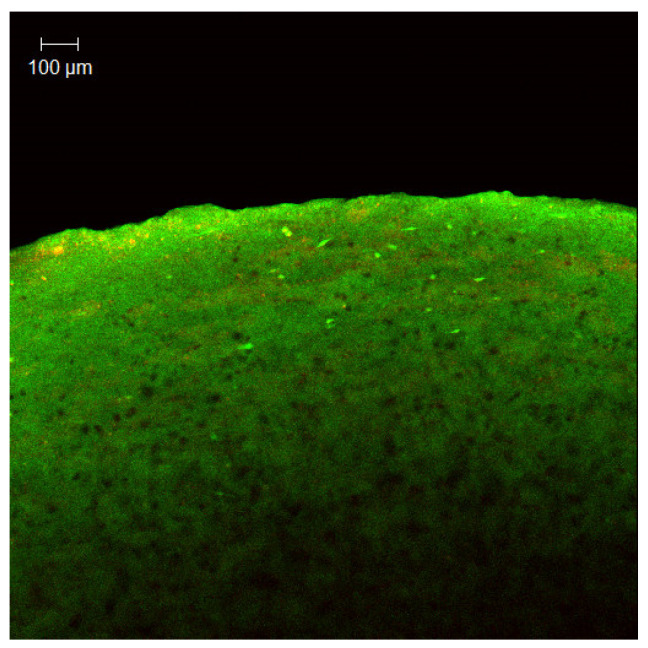
Confocal microscopy image of the surface of the 3D oral mucosal model.

Figure 4 shows the confocal microscopy image of the 3D oral mucosal model. The results of the confocal microscopy analysis were consistent with the histological and OCT findings and showed similar patterns in the different test groups.

### 3.5. Inflammatory Cytokine Release

**Figure 5:**
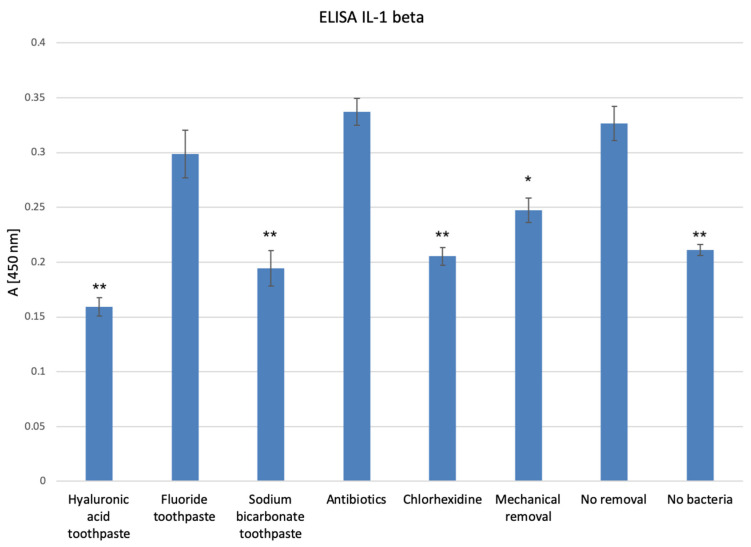
IL-1β release from the 3D oral mucosal models in different test groups. *, statistically significant difference (*p* < 0.05) in IL-1β release in the mechanical removal group compared with the no-removal group. **, statistically significant difference (*p* < 0.01) in IL-1β release in the hyaluronic acid toothpaste, sodium bicarbonate toothpaste, chlorhexidine mouthwash, and no-bacteria groups compared with the mechanical bacterial removal group.

Addition of oral bacteria to the 3D oral mucosal models significantly increased the amount of IL-1β inflammatory cytokine released into the culture medium (*p* < 0.05). ELISA analysis of the tissue culture supernatants detected a statistically significant reduction (*p* < 0.05) in the quantities of IL-1β inflammatory cytokines released from the inflamed 3D oral mucosal models treated with a hyaluronic acid toothpaste, a sodium bicarbonate toothpaste, and a chlorhexidine mouthwash compared with mechanical bacterial removal only (Figure 5).

**Figure 6:**
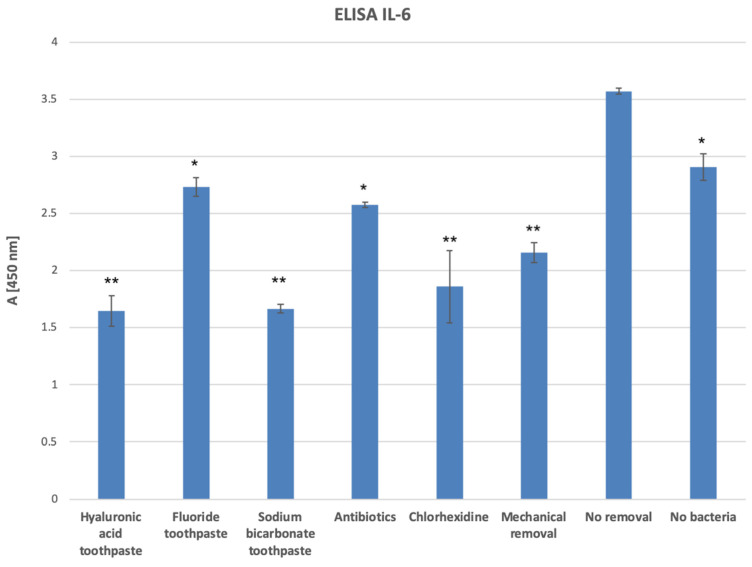
IL-6 release from the 3D oral mucosal models in different test groups. *, statistically significant difference (*p* < 0.05) in IL-6 release in the fluoride toothpaste, antibiotic, and no-bacteria groups compared with the no-removal group. **, statistically significant reduction (*p* < 0.01) in IL-6 release in the hyaluronic acid toothpaste, sodium bicarbonate toothpaste, chlorhexidine mouthwash, and mechanical removal groups compared with the rest of the groups.

ELISA analysis of the tissue culture supernatants also detected a similar cytokine release pattern for the inflammatory marker IL-6 released from the 3D oral mucosal models into the culture media in different test groups (Figure 6).

**Figure 7:**
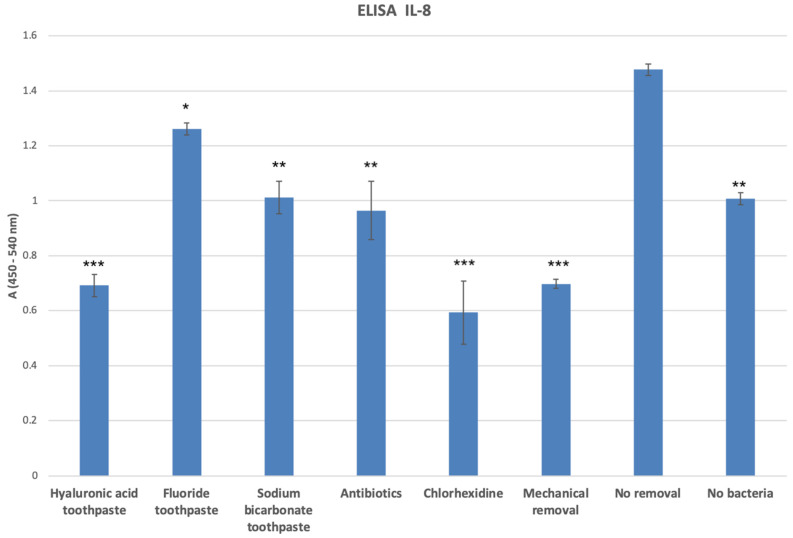
IL-8 release from the 3D oral mucosal models in different test groups as assessed by ELISA. *, statistically significant difference (*p* < 0.05) in IL-8 release in the fluoride toothpaste group compared with the no-removal control group. **, statistically significant difference (*p* < 0.01) in IL-8 release in the sodium bicarbonate toothpaste, antibiotic, and no-bacteria groups compared with the rest of the groups. ***, statistically significant reduction (*p* < 0.001) in IL-8 release in the hyaluronic acid toothpaste, chlorhexidine mouthwash, and mechanical removal groups compared with the rest of the groups.

As demonstrated in Figure 7, addition of oral bacteria to the 3D oral mucosal models increased the amount of IL-8 inflammatory cytokine released into the culture medium. All bacterial removal methods resulted in a reduction in the amount of IL-8 released from the oral mucosal models. The differences were statistically significant (*p* < 0.05).

### 3.6. Anti-Bacterial Testing

#### 3.6.1. PrestoBlue Assay

**Figure 8:**
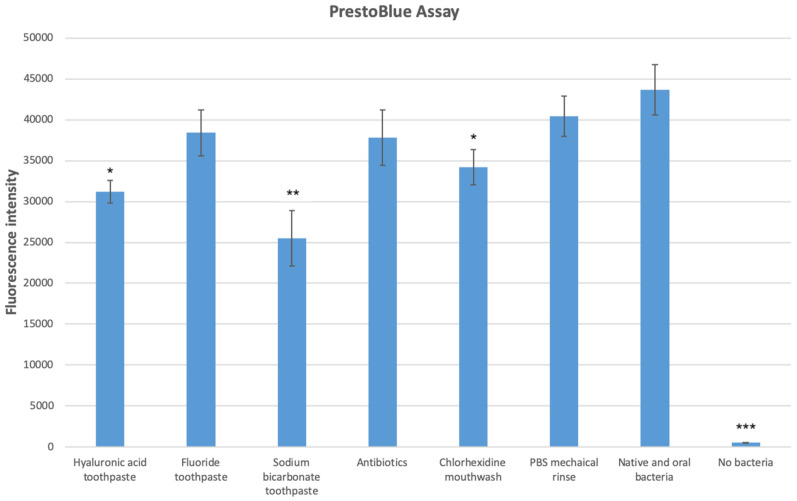
Viability of saliva bacteria cultured on non-vital mucosal matrices following treatment with different test agents as assessed by the PrestoBlue assay. *, statistically significant difference (*p* < 0.05) in bacterial viability in the hyaluronic acid toothpaste and chlorhexidine mouthwash groups compared with the control group. **, statistically significant reduction (*p* < 0.01) in bacterial viability in the sodium bicarbonate toothpaste group compared with the control and other groups. ***, statistically significant difference (*p* < 0.001) in the viability of the no-bacteria group compared with all other groups.

The results of the PrestoBlue assay for the effects of different test agents on the viability of saliva bacteria cultured on non-vital mucosal matrices are presented in Figure 8. Treatment with the sodium bicarbonate toothpaste, hyaluronic acid toothpaste, and chlorhexidine mouthwash caused the largest reduction in the viability of oral bacteria cultured on non-vital mucosal matrices (*p* < 0.05) compared with the other test agents.

#### 3.6.2. Confocal Microscopy

The results of confocal microscopy analysis of saliva bacteria cultured on non-vital mucosal matrices were also consistent with the findings of the viability testing in different test groups and showed a similar pattern.

## 4. Discussion

In vitro and ex vivo 3D models of an oral mucosa have been employed for the modeling of oral diseases and studying the mechanisms of oral bacterial and fungal infections [17]. In this study, an inflamed 3D tissue-engineered oral mucosa model was developed consisting of human oral epithelial cells, oral fibroblasts, and THP-1 monocytes. Cell lines were used to enhance the reproducibility of the models and eliminate batch-to-batch variations when primary cells from different patients are used. The incorporation of immunocompetent THP-1 monocytes [18] into the model enabled the detection of the inflammatory response to different stimuli, such as LPS and oral bacteria. Other studies have attempted to produce immunocompetent tissue-engineered oral mucosa models by adding the Langerhans-like cell line MUTZ-3 to the reconstructed human gingival epithelial layer [1]. Although THP-1 monocytes have been used in monolayer cell culture systems to investigate the biocompatibility of dental materials [19,20], this was the first study to use a 3D full-thickness tissue-engineered human oral mucosal model containing both THP-1 monocytes and oral saliva bacteria to visualize and evaluate the anti-inflammatory and anti-bacterial effects of different oral care products.

In this study, we used a combination of qualitative and quantitative approaches to evaluate the biological endpoints. Histology helps us visualize and assess any morphological changes in the epithelial and connective tissue layers of the oral mucosal model. The alterations in the oral mucosa can also be visualized by optical coherence tomography (OCT) and confocal microscopy, which are non-invasive imaging methods and enable the assessment of the superficial layers of the 3D oral mucosa tissue model and bacterial biofilm without the need for histological processing. This approach has the potential to be used in the clinic to assess and monitor mucosal soft tissue pathology and obviate the need for invasive tissue biopsy procedures [21].

The PrestoBlue assay was used to assess the viability of the 3D tissue models. PrestoBlue is a biocompatible dye and can be used to continuously monitor tissue viability and proliferation. In addition, changes in its color can be detected by measuring the fluorescence, which provides higher detection sensitivity compared with other cell viability assays [22].

The inflammatory markers investigated in this study included IL-1β, IL-6, and IL-8.

IL-1 is a group of 11 cytokines involved in the regulation and initiation of inflammatory responses [23]. IL-1α and IL-1β are the most-studied cytokines, as they were discovered first and possess strong pro-inflammatory effects.

Interleukin-8 is an important inflammatory mediator and plays a crucial role in neutrophil recruitment and degranulation [24]. Its secretion is increased by oxidative stress leading to the recruitment of inflammatory cells, making IL-8 a key factor in localized inflammation.

On the other hand, Interleukin-6 has both pro-inflammatory cytokine properties and an anti-inflammatory myokine function. IL-6 plays a role in stimulating the inflammatory and auto-immune processes in many conditions, including diabetes, cancer, and auto-immune diseases [25].

From the oral biocompatibility and anti-inflammatory perspectives, these cytokines (especially IL-1β) are the most clinically relevant cytokines when testing oral care products as they have been directly associated with gingivitis and periodontal diseases [26].

Our previous study showed major differences in cytokine expression profiles between infected and non-infected oral mucosal models using both monolayer and 3D cell culture systems [27]. In addition, other studies have also reported a significant increase in the quantities of inflammatory markers released from 3D oral mucosal models in response to exposure to candida albicans [28,29,30].

The effects of the addition and removal of oral bacterial biofilms on tissue-engineered human oral mucosal models were investigated in this study using different bacterial removal methods. Since a pipette was used to rinse the tissue with PBS, the term “mechanical rinse” was used in this case. Multiple-endpoint analyses of the infected oral mucosal models treated with different anti-bacterial agents showed consistent outcomes in terms of tissue viability (in vital 3D models), histology, OCT, and confocal microscopy findings.

It is important to note that the first PrestoBlue assay reported the cumulative viability of the oral bacteria and the cells within the oral mucosal models since bacteria themselves have metabolic activity and contribute to the overall viability within the infected groups. The ranking of the test groups in terms of combined tissue and bacterial viability was as follows:

No removal > mechanical rinse > antibiotics > fluoride toothpaste > sodium bicarbonate toothpaste > hyaluronic acid toothpaste > chlorhexidine mouthwash

The results of the PrestoBlue assay of bacterial cultures on non-vital mucosal matrices showed that the ranking in terms of bacterial viability was as follows:

No removal > mechanical rinse > antibiotics = fluoride toothpaste > chlorhexidine mouthwash > hyaluronic acid toothpaste > sodium bicarbonate toothpaste.

These findings indicate that treatment with the chlorhexidine mouthwash may have reduced the viability of the cells within the oral mucosal models as well as the bacterial cultures. This was also evident in the histological images, where 3D oral mucosal models treated with the chlorhexidine mouthwash had a very thin epithelial layer and a reduced number of fibroblasts within the connective tissue layer. A possible reason for this observation is the fact that 0.2% *w*/*v* chlorhexidine digluconate mouthwash was used in its pure form as it was not possible to produce an isotonic solution of the mouthwash in PBS as it would have altered the chlorhexidine’s concentration. A hypotonic solution could potentially reduce the viability of the cells within the 3D oral mucosal models in addition to the direct toxic effect of chlorhexidine on the vital cells. These findings are consistent with a previous study that reported that chlorhexidine gluconate (0.05%) was uniformly toxic to both cultured human cells and microorganisms [31]. The cytotoxicity of chlorhexidine mouthwash to human oral fibroblasts and keratinocytes has also been reported in other studies [32,33].

Regarding the effects of toothpastes on the viability of oral mucosal cells, it has been reported that that the ingredients used in toothpastes, especially the detergent used in toothpastes, can be associated with changes in the in vitro cell viability [34]. Other studies have also reported variations in the biocompatibility and anti-bacterial effects of different toothpastes using in vitro monolayer cell culture systems [35].

In terms of anti-inflammatory testing, the positive control group (no-removal group) showed the highest level of inflammation compared with the other groups. The negative control group (no bacteria) showed some inflammation, as the 3D model itself can produce cytokines without the presence of bacteria. Depending on the anti-bacterial and anti-inflammatory potential of the test groups, different levels of inflammation could be observed in the other test groups. Treatment with the hyaluronic acid toothpaste and the sodium bicarbonate toothpaste resulted in the greatest reduction in the bacteria-induced release of the pro-inflammatory cytokines IL-1β, IL-6, and IL-8 from the 3D oral mucosal models. The hyaluronic acid toothpaste was more effective in reducing the release of IL-1β and IL-8 than the Corsodyl toothpaste.

Although at the clinical level it is well-known that chlorhexidine mouthwash is more effective in reducing the quantities of oral bacteria compared with other oral care products, the results of this in vitro study show that both hyaluronic acid and sodium bicarbonate were more effective than chlorhexidine mouthwash in reducing the quantities of IL-6 and IL-1β inflammatory cytokines and in reducing the viability of oral bacteria. These observations could be due to the differences in the biological systems and exposure protocols used in different studies and would be an area for further investigation in the future.

The histological examination showed that 3D oral mucosal models treated with the hyaluronic acid toothpaste and sodium bicarbonate toothpaste had thinner epithelial layers compared with the control group. These findings are consistent with a previous study by Mostefaoui et al. that used tissue-engineered oral mucosal models to assess the biocompatibility of different dentifrices. It was reported that toothpastes (Aquafresh and Crest) contributed to epithelial desquamation, which was substantial at 24 h of contact but was limited to the superficial layers of the oral mucosal models. Tissue cell death was not increased, suggesting that the toothpastes had accelerated the desquamation of only differentiated epithelial cells at the surface of the 3D oral mucosal models. It was also concluded that the toothpastes contributed to the modulation of the inflammatory process as assessed by the release of IL-1β, IL-8, and TNF-α [36].

## 5. Conclusions

From the results of this study, it can be concluded that the incorporation of THP-1 monocytes into tissue-engineered human oral mucosa models enabled the visualization and quantification of LPS-induced and bacteria-induced inflammation in the 3D oral mucosa models. Multiple-endpoint analyses using a PrestoBlue tissue viability assay, histological examination, non-invasive OCT imaging, confocal microscopy, and ELISA measurement of pro-inflammatory cytokine release demonstrated that the 3D oral mucosal models were more clinically relevant and more informative than the 2D cell culture test models that are commonly used. The inflamed 3D oral mucosal model developed in this study has the potential to be used as a suitable in vitro model for testing the biocompatibility, anti-inflammatory properties, and anti-bacterial properties of oral care products, including mouthwashes and toothpastes. The results of this study indicate that the chlorhexidine mouthwash had both anti-bacterial and cytotoxic effects on the 3D oral mucosal model. The hyaluronic acid toothpaste and sodium bicarbonate toothpaste had significant anti-bacterial and anti-inflammatory effects on the 3D oral mucosal model and may cause exfoliation of the superficial desquamated layers of the oral mucosa epithelium.

## Data Availability

The original contributions presented in the study are included in the article, further inquiries can be directed to the corresponding author.
